# Comparative Genome Analysis Reveals the Molecular Basis of Niche Adaptation of *Staphylococcus epidermidis* Strains

**DOI:** 10.3389/fgene.2020.566080

**Published:** 2020-11-09

**Authors:** Fei Su, Rui Tian, Yi Yang, Hexin Li, Gaoyuan Sun, Ying Li, Bingqing Han, Xiaomao Xu, Xue Chen, Gang Zhao, Hongyuan Cui, Hongtao Xu

**Affiliations:** ^1^Clinical Biobank, Beijing Hospital, National Center of Gerontology, Institute of Geriatric Medicine, Chinese Academy of Medical Sciences, Beijing, China; ^2^Department of Cardiovascular Disease, Beijing Anzhen Hospital, Capital Medical University, Beijing, China; ^3^Department of Otorhinolaryngology, Beijing Hospital, National Center of Gerontology, Institute of Geriatric Medicine, Chinese Academy of Medical Sciences, Beijing, China; ^4^The Key Laboratory of Geriatrics, Beijing Hospital, National Center of Gerontology, Institute of Geriatric Medicine, Chinese Academy of Medical Sciences, Beijing, China; ^5^Department of Laboratory Medicine, Beijing Hospital, National Center of Gerontology, Institute of Geriatric Medicine, Chinese Academy of Medical Sciences, Beijing, China; ^6^Department of General Surgery, Beijing Hospital, National Center of Gerontology, Institute of Geriatric Medicine, Chinese Academy of Medical Sciences, Beijing, China

**Keywords:** *Staphylococcus epidermidis*, antimicrobial resistance, pan-genome, mobile genetic element, comparative genome analysis

## Abstract

*Staphylococcus epidermidis* is one of the most commonly isolated species from human skin and the second leading cause of bloodstream infections. Here, we performed a large-scale comparative study without any pre-assigned reference to identify genomic determinants associated with the diversity and adaptation of *S. epidermidis* strains to various environments. Pan-genome of *S. epidermidis* was open with 435 core proteins and had a pan-genome size of 8,034 proteins. Genome-wide phylogenetic tree showed high heterogeneity and suggested that routine whole genome sequencing was a powerful tool for analyzing the complex evolution of *S*. *epidermidis* and for investigating the infection sources. Comparative genome analyses demonstrated a range of antimicrobial resistance (AMR) genes, especially those within mobile genetic elements. The complicated host-bacterium and bacterium-bacterium relationships help *S*. *epidermidis* to play a vital role in balancing the epithelial microflora. The highly variable and dynamic nature of the *S. epidermidis* genome may contribute to its success in adapting to broad habitats. Genes related to biofilm formation and cell toxicity were significantly enriched in the blood and skin, demonstrating their potentials in identifying risk genotypes. This study gave a general landscape of *S. epidermidis* pan-genome and provided valuable insights into mechanisms for genome evolution and lifestyle adaptation of this ecologically flexible species.

## Introduction

The coagulase-negative *Staphylococcus epidermidis* is a common human skin commensal bacterium that can be cultured from the body surface of almost all healthy individuals. It also plays a central role in the skin microbiome (Otto, 2009; Oh et al., 2016; Zhou et al., 2020), especially in keeping the ecological balance of human skin microflora (Schommer and Gallo, 2013). *S*. *epidermidis* can produce various bacteriocins, which directly kill other microorganisms and may enhance survival of the producer strains in a competitive fashion (Jetten and Vogels, 1972; Jack et al., 1995). Especially, serine protease Esp, secreted by *S. epidermidis*, can inhibit the biofilm formation of *Staphylococcus aureus* and destroy the pre-existing *S. aureus* biofilms (Iwase et al., 2010).

However, *S. epidermidis* is the second most common cause of nosocomial infections, which in most cases are antibiotic-resistant (Otto, 2009; Joo and Otto, 2015). Antibiotic resistance remarkably complicates the treatment and increases the medical expenses (Foster, 2017; Lee et al., 2018). The large gene pool of antibiotic resistance in *S. epidermidis* is shared with many other pathogenic species (e.g., *S. aureus*) through rare horizontal gene transfer events (Diep et al., 2006). Mobile genetic elements such as multidrug-resistant conjugative plasmids, arginine catabolic mobile element (ACME; Diep et al., 2006), and staphylococcal chromosome cassette *mec* (SCC*mec*) elements (Mcmanus et al., 2015) conferring β-lactam resistance are transferred frequently, enabling rapid evolution and adaptation of *S. epidermidis* against antibiotic selection pressure (Miragaia et al., 2007; Bloemendaal et al., 2010). When the protective layer of the human epithelium is breached and the host immunity fails, staphylococcal infections can become extremely dangerous and even fatal (Yao et al., 2005). *S. epidermidis* is particularly associated with the increased use of indwelling medical devices such as artificial heart valves, prosthetic joints, and vascular catheters, which provide a substrate for biofilm formation. On the other hand, during the long-time coevolution with various pathogens, human beings have developed versatile immunity system with antimicrobial peptides (AMPs) as the first line of innate immune defense on the human skin (Zhou et al., 2020); meanwhile, *S. epidermidis* also owns multiple mechanisms such as surface charge alteration, extracellular proteases, exopolymers, and efflux pump proteins to fight against AMPs (Joo and Otto, 2015). The complex host-bacterium and bacterium-bacterium relationships make it necessary to investigate the genetic diversity, genome evolution, and lifestyle adaptation of *S. epidermidis*.

Much attention has been focused on understanding the evolution and spread of *S. epidermidis* by different methods (Miragaia et al., 2007; Meric et al., 2015, 2018; Zhou et al., 2020). As high-throughput sequencing has yielded a large amount of genomic data on *S. epidermidis*, it is essential to perform more comprehensive comparative and evolutionary studies on ecologically diverse strains of *S. epidermidis*. Here, we compared the genomic features of *S. epidermidis* isolates of clinical and non-clinical relevance by using a pan-genome analysis of 198 publicly available *S. epidermidis* strains at the GenBank database of National Center for Biotechnology Information (NCBI) with isolation or clinical information when the analysis was performed. We assembled the consensus “pan-chromosome” without any pre-assigned genome reference and identified both core and variable regions within the chromosome. Second, we utilized a comparative genomics approach on 198 genomes to analyze the diversity of antibiotic resistance of *S. epidermidis*. Our results revealed that *S. epidermidis* isolates encoded a vast collection of genetic determinants and mechanisms to confer antibiotic resistance, AMPs resistance, and survival adaptations. These analyses were expected to provide insight into the coevolution of *S. epidermidis* as a nosocomial pathogen and directly aid the future efforts for large-scale epidemiological studies of this continuously evolving multi-drug resistant organism.

## Materials and Methods

### Strains

A total of 198 *S*. *epidermidis* isolates were selected to represent known diversity within geographical position, isolation source, and host tissue sampled when this analysis was performed, including reference genome of strain RP62A (Gill et al., 2005). All the available genome sequence of *S. epidermidis* strains and related annotation data were downloaded through the GenBank database (Benson et al., 2013) of NCBI (see Supplementary Table S1 in the Supplementary Material). Totally 121 strains were isolated from different human tissues and the others were from environments.

### SCC*mec* and ACME Typing

An SCC*mec* sequence cassette database was prepared with the following accession numbers downloaded from NCBI: AB033763.2 (Type I), AB433542.1 (Type I.2), D86934.2 (Type II), AB261975.1 (Type II.4), AJ810123 (Type II-B), AB127982.1 (Type II-B), AM983545.1 (Type II-D), HE858191.1 (Type II-E), AB037671.1 (Type III), HM030721.1 (Type IV), HM030720.1 (Type IV), AM292304.1 (ZH47 mobile elements), AB425824.1 (Type IV), EU437549.2 (Type IV-A), AB063172.2 (Type IV-A), AB063173 (Type IV-B), AY271717.1 (Type IV-C), AB096217 (Type IV-C), AB245470.1 (Type IV-C), AB097677.1 (Type IV-D), AJ810121.1 (Type IV-E), DQ106887.1 (Type IV-G), AB633329.1 (Type IV-I), AB425823.1 Type IV), AB121219.1 (Type V), AB478780.1 (Type V), AB512767.1 (Type V), AF411935.3 (Type VI), AB462393.1 (Type VII), AB373032.1 (Type V-C1), FJ670542.1 (Type VIII), FJ390057.1 (Type VIII), AB505628.1 (Type IX), AB505630.1 (Type X), and FR821779.1 (Type XI; Ugolotti et al., 2016).

The ACME-*arc*A and ACME-*opp*3AB genes were used as markers of the ACME-*arc* cluster and the ACME-*opp*3 cluster, respectively. ACME was classified as type I (contains the ACME-*arc*A and ACME-*opp*3AB gene clusters), type II (carries only the ACME-*arc*A locus), and type III (carries only the ACME-*opp*3AB locus; Barbier et al., 2011). ACME-*arc*A and ACME-*opp*3AB identified in this study were compared with the reference sequences of ACME-*arc*A (USA300_FPR3757) and ACME-*opp*3AB (USA300_FPR3757).

### kSNP *S. epidermidis* Trees

Because microbial genomes are subject to massive gene gains and losses, insertion, deletions and rearrangements, alignment of whole microbial genome sequences has proven to be computationally intensive. Here, we used kSNP (version: 3.0^1^), a validated method to build the phylogenetic tree without alignment (Gardner et al., 2015) by using a *k*-mer length 19 nucleotides and based on a requirement that at least 80% of the genomes have a nucleotide at a given Single-nucleotide polymorphisms (SNPs) position in order for the SNP to be considered to be a core and included in tree building. A total of 1,832 core SNP positions were identified. jModelTest2 was used to carry out statistical selection of best-fitmodels of nucleotide substitution by implementing different model selection strategies (Ehsan Vafadarnejad et al., 2020) and maximum-likelihood tree was built with RAxML (Stamatakis, 2014) with generalized time reversible (GTR) model. Branch support values were inferred from a rapid bootstrap method applied with 100 replications. Visualization and annotation of the phylogenetic tree was performed by ggtree (Yu et al., 2018).

### Pan-Genome Analysis

Cluster of orthologous proteins were generated with version 3.24 of PanOCT^2^) as previously described (Fouts et al., 2012). Briefly, PanOCT dealt with recently diverging paralogs by using neighborhood gene information. All the parameters were set to default values except for the length ratio to discard shorter protein fragments when a protein was split due to a frameshift or other mechanisms was set to 1.33 as recommended by the authors. Orthologous clusters were stringently defined as all sequences in a cluster having shared sequence identity ≥ 70% and coverage ≥ 75%. Plots and calculations of pan-genome sizes, new genes discovered, and pan-genome status were also determined as described previously (Tettelin et al., 2005). Core genes were defined as the genes present in all of the *S*. *epidermidis* genomes and accessory genes as genes present in at least one isolate.

### Characterization of Strains

*In silico* multilocus sequence typing of 198 strains was performed with the MLST 1.8 online server (Larsen et al., 2012). The antimicrobial resistance (AMR) genes in the sequenced isolates were identified by BLASTp (Altschul et al., 1990) searching against the databases of ARDB (Liu and Pop, 2009), CARD(Alcock et al., 2020), and ResFinder (Zankari et al., 2012), with 10^–5^ as expect value threshold. Genes conferring virulence factors were identified using BLASTp with VFDB (Chen et al., 2012). Given that many virulence factors for *S*. *epidermidis* that are not contained in the VFDB, we used the orthologous proteins and virulence factors from RP62a (Assembly: GCF_000011925.1; Gill et al., 2005) and ATCC1228 (Assembly: GCA_000007645.1; Zhang et al., 2003) to make up the missing information.

### Functional Analysis

All genes were BLASTed against the database of KOBAS 2.0^3^ (Xie et al., 2011). The cutoffs were BLASTp *E*-value < 10^–5^ and BLAST subject coverage > 70%. We used the genes from the same genome as the default background distribution and considered the only pathways for which there were at least two genes mapped. For the enrichment analysis, Fisher’s exact test was performed, and Bonferroni correction was used to reduce the high overall Type-I error with p.adjust from R package.

### Statistical Analyses

The differences in the prevalence of AMR genes and phenotypes among isolates were analyzed by using two-tailed Fisher’s exact test, and Bonferroni correction was also performed as mentioned above. All the statistical analyses were carried out using R package (version: 3.3). A *P*-value of < 0.05 was regarded as statistically significant.

## Results

### Core Pan-Genome of *S. epidermidis*

**FIGURE 1:**
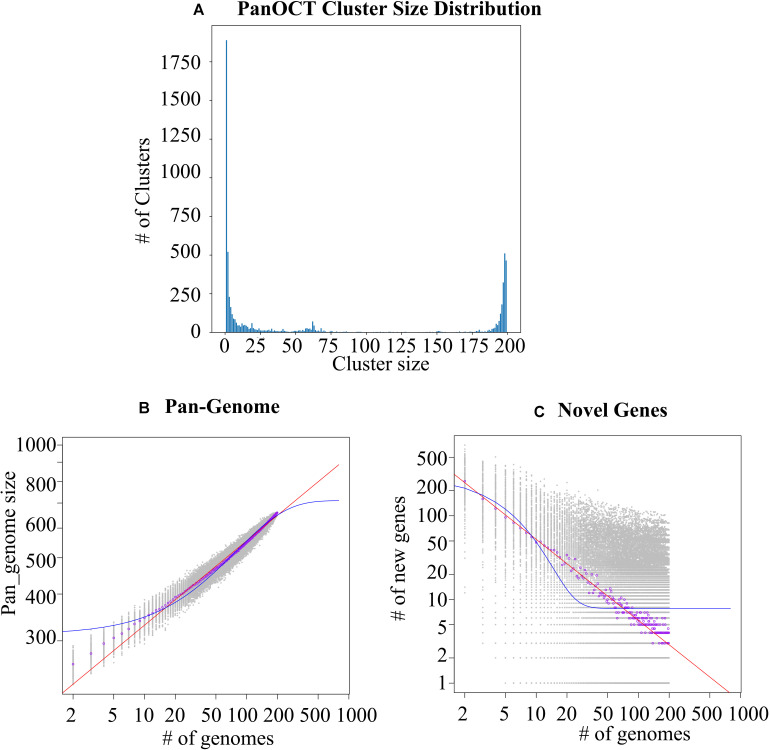
Analysis of the *Staphylococcus epidermidis* pan-genome. **(A)** The distribution of protein cluster sizes generated from the comparison of 198 *S. epidermidis* genomes using PanOCT. **(B)** The pan-genome size (left) and the number of novel genes discovered with the addition of each new genome (**C**, right) were estimated for all 198 genomes using a pan-genome model based on the original Tettelin et al. (2005) model.

Despite the intensive effort to characterize *S. epidermidis* and the sizable number of whole genome comparisons in literature (Conlan et al., 2012; Meric et al., 2018), more and more genome data is rapid accumulated and could easily obtained from public database, such as NCBI. Based on PanOCT, a total of 8,034 orthologous protein clusters were identified from a collection of all *S. epidiermidis* genomes publicly available at the time of the analysis (Supplementary Table S1). PanOCT only includes non-paralogs in clusters and uses conserved gene neighborhood to separate duplicated genes. This means that insertion sequence elements that are in novel contexts will often form singleton clusters even though they are identical in sequence to other IS elements within or between genomes analyzed. When the “core” pan-genome was defined to be present at all 198 genomes analyzed, there were 435 (5.4%) core protein clusters and 2,915 (36.3 %) novel clusters (groups with a single member from a single genome; Figure 1A). To predict the theoretical maximum pan-genome size (i.e., the total number of genes, including core, unique, and accessory genes), a pan-genome model was implemented using medians and an exponential decay function (Figure 1B). The maximum pan-genome size was estimated to be 12,554 ± 65 genes. To determine whether the *S. epidiermidis* pan-genome had an unlimited large gene repertoire (open) or seemed to be limited by a maximum number of genes in their gene pool (closed), the number of new genes identified (i.e., unique or strain-specific genes) for each genome added was determined and fit to a power law function (n = κ N^–α^) as described previously (Tettelin et al., 2005). According to the result, we found the pan-genome of *S. epidiermidis* appeared to be open (α = 0.226 ± 0.002; Figure 1B). For each genome added, the number of new genes was extrapolated by calculating tg(θ), which was determined to be 7.7 ± 0.4 (Figure 1C).

**FIGURE 2:**
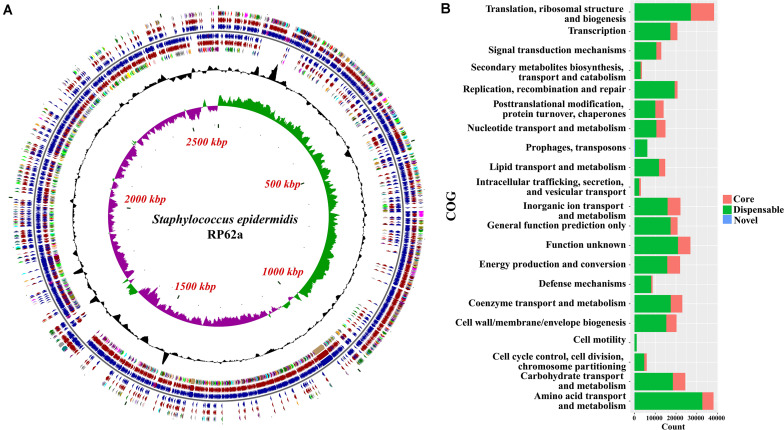
Functional analysis of the pan-genome of *Staphylococcus epidermidis*. **(A)** Distribution of core/accessory/novel genes in the type strain RP62a. Starting from the outermost ring the feature rings depict: (1) clusters of orthologous group (COG) functional categories for forward strand coding sequences; (2) Core (brown)/accessory (blue) genes for forward strand coding sequences; (3) Forward strand sequence features; (4) Reverse strand sequence features; (5) Core (brown)/accessory (blue) genes for reverse strand coding sequences; (6) COG functional categories for reverse strand coding sequences. (7) GC content; (8) GC skew. The colors of different COG functional categories were following the definition of Grant et al. (2012). **(B)** Numbers of core, accessory and novel genes for each clusters of orthologous group (COG) category. COGs significantly enriched (adjusted *P*-value < 0.05, Fisher exact test) in core, accessory or novel genes are marked with red asterisk.

The function of the genes within the variable genome was investigated by assigning all gene clusters to clusters of orthologous groups (COGs; Tatusov et al., 2003), and the results showed that novel genes were most likely to be assigned to categories (Figure 2 and Supplementary Tables S2, S3) such as mobilome, ribosomal structure and biogenesis, carbohydrate transport and metabolism, and nucleotide transport and metabolism, based on the result of Fisher’s exact test.

### Phylogenetic Relationship of *S. epidermidis* Isolates

**FIGURE 3:**
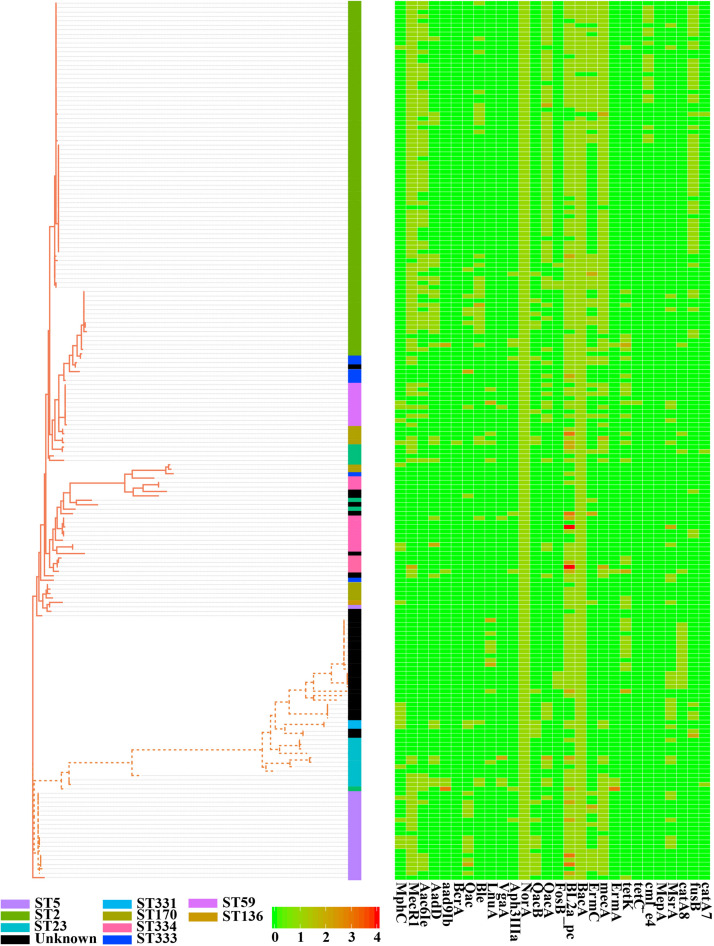
Phylogenetic single-nucleotide polymorphism (SNP) tree of *Staphylococcus epidermidis* strains. A whole-genome core SNP maximum likelihood tree was constructed for 198 genomes with kSNP and RAxML. Heatmap on the right indicates copies of 28 genes involved in antibiotic resistance. Legends on the bottom stand for copy number of resistant genes. The scale bar, which is an indicator of genetic distance, indicates 0.01.

To estimate the genetic relationships among *S. epidermidis* strains, we compared all 198 genomes by using a single nucleotide polymorphism-based phylogeny. SNPs were identified from the combined set of genome sequences by using kSNP. Nucleotide positions present in at least 80% of all genomes were used to build a Maximum-Likelihood phylogenetic tree with RAxML following the tutorial. Strikingly, the 198 *S. epidermidis* isolates formed two distinct groups (Figure 3), called Cluster A (solid line) and B (dotted line). Most of Sequence Type (ST) 2 nosocomial isolates were near-identical at the nucleotide level for all core genes (Supplementary Table S4). All of ST 2 strains in this study presented in Cluster A and had an extremely short evolutionary distance from each other (scaled branch lengths < 0.01), with 99.7% identical in sequence, indicating that these strains were probably derived from a recent common ancestor. By contrast, Cluster B represented a lineage with reduced virulence and all of ST 5 commensal strains presented in Cluster B and clustered together. The rest of Cluster B had a much longer evolutionary distance from ST 5 strains. This clade might have a more complex history of evolution and produce a variety of sub-groups.

### Antimicrobial Resistance Across *S. epidermidis*

Antimicrobial resistance is very common among *S. epidermidis* isolates and often limits treatment options (Kleinschmidt et al., 2015). Given the clinical importance of AMR in *S. epidermidis*, we performed a genome-wide analysis of all known AMR genes within our genomic dataset. According to the analysis of ARDB, ResFinder and CARD databases, we found 28 different genes involved in 31 antibiotics (Figure 3). Nearly all isolates carried at least one antibiotic resistance gene. Among the genes involved in AMR, our data showed that there were two genes, *nor*A (AAW53745.1) and *bac*A (AAW53717.1), conserved in all strains. Based on the enrichment analysis of strains from different niches, we found that strains from different sources (skin, blood, environment, and plant) had significantly different antibiotic resistance profiles: isolates from blood (9 antibiotic resistance genes) and skin (8 antibiotic resistance genes) had significantly enriched antibiotics (Supplementary Table S5), while isolates from environment had no significantly enriched antibiotics. First-line antibiotic therapy for catheter-related bloodstream infections was vancomycin. None of these isolates were resistant to the antibiotic at the genetic level, regardless of isolation source.

### SCC*mec* and ACME in *S. epidermidis*

Staphylococcal chromosome cassette *mec*, or *staphylococcal* cassette chromosome *mec*, is a mobile genetic element that carries the central determinant for broad-spectrum beta-lactam resistance encoded by the *mec*A gene (AAW53314.1), a mobile genetic element of *Staphylococcus* bacterial species (IWG-SCC, 2009; Mcmanus et al., 2015). According to the completeness of genome in this study (only 7 complete genome sequences), we only analyzed the genes from well-defined SCC*mec* genomic islands (Kos et al., 2012). There were 58.6% (116/198) of *S. epidermidis* strains, in which complete *mec* gene complexes, *mec*A (AAW53314.1), and *mec*R1 (AAW53313.1) genes were detected (Supplementary Table S1). However, only 39.4% (78/198) of strains had *ccr* gene complex from type IV cassette, in which both *ccr*A and *ccr*B were present but not in RP62a. Similar to the previous results (Conlan et al., 2012; Du et al., 2013), nearly all of the ST 2 nosocomial isolates (94.6%, 70/74) had at least one copy of *mec*A from type IX cassette and *mec*R1 from Type VIII or IV-G cassette. On the other hand, a high prevalence (98%, 195/198) of ACME was found in *S. epidermidis* strains in this study, of which 22.7% (45/198) was type I and 75.8% (150/198) was type II.

### Biofilm Formation of *S. epidermidis*

**FIGURE 4:**
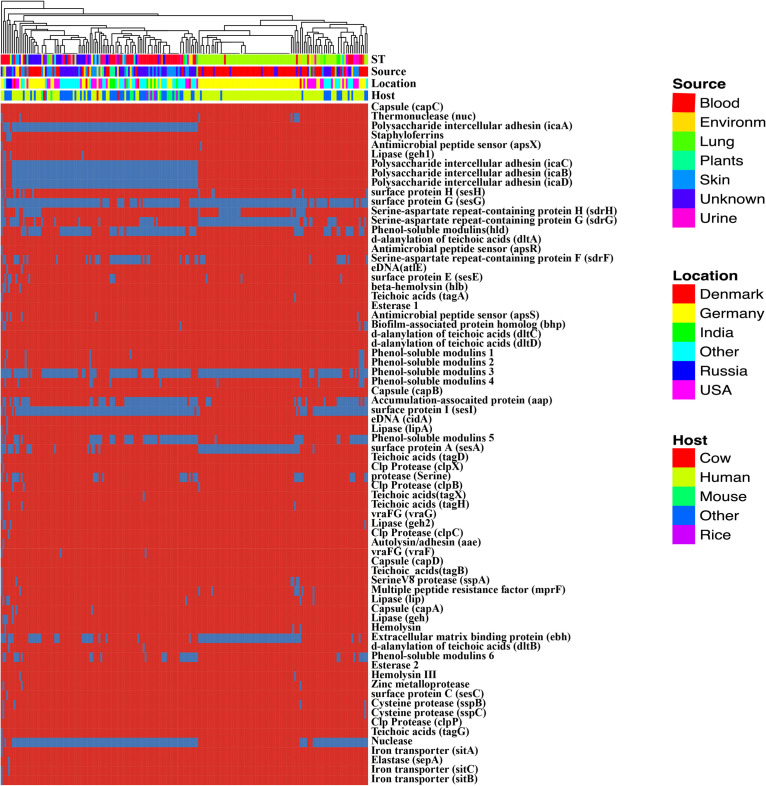
Heatmap of virulence factors among the *Staphylococcus epidermidis* strains. The dendrogram was generated using complete linkage clustering of copies of genes involved in virulence factors. The red color stands for genes that exist in the genomes and the blue color for missing ones. Legends on the right stand for colors of different host, isolates and geographic information.

Biofilm formation is the major of virulence factor of *S. epidermidis* strains, which contributes to the persistence of clinical infection. Here, we analyze some well-known factors involved in biofilm formation such as adhesive molecules, including polysaccharide intercellular adhesin (*ica*ABCD), proteinaceous factors (*bhp* (AAW53225.1), and *aap* (AAW53239.1), teichoic acids, extracellular DNA (Supplementary Table S6). The polysaccharide intercellular adhesion (*ica*ABCD) genes that encode biofilm-associated genes for poly-*N*-acetylglucosamine synthesis were found in 60% of the commensal isolates, in agreement with previous studies (Du et al., 2013). Especially, any of the *ica* genes was not found in some ST 2 strains (Figure 4). Gene *aap* was enriched in the blood (adjusted *P*-value < 0.01) compared to the remaining isolates and therefore might be a potential biomarker for *S. epidermidis* infection. We analyzed the enrichment of all genes involved in virulence factors and found the *ica*ABCD was significantly enriched despite the sources or sequence types.

### Human-Bacterium and Bacterium-Bacterium Interactions in *S. epidermidis*

**FIGURE 5:**
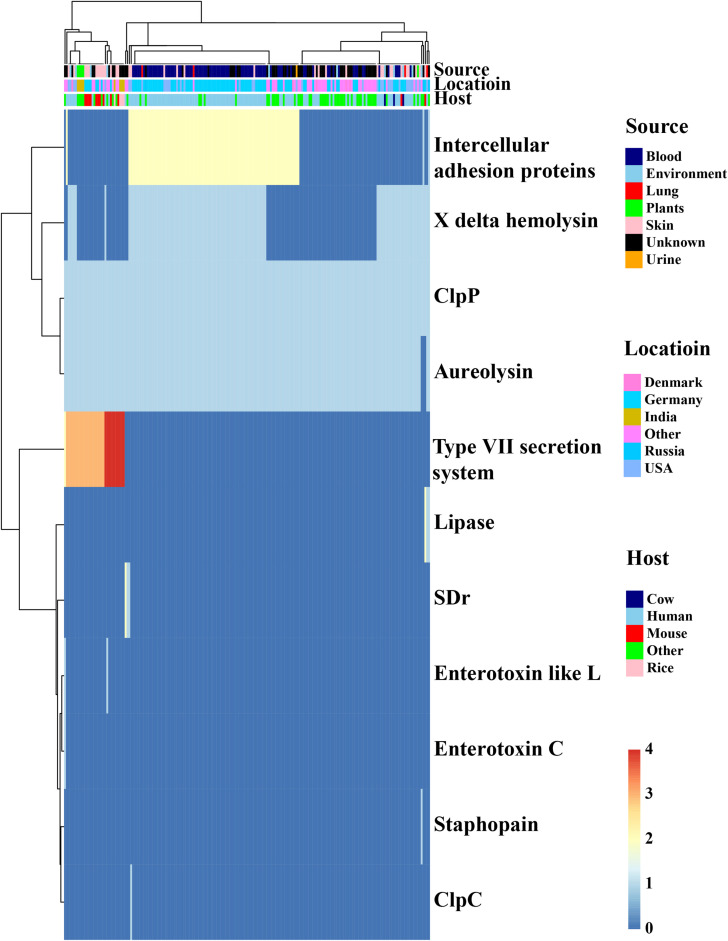
*In silico* analysis of virulence factors of the *Staphylococcus epidermidis* strains. The types of virulence factors were following the VFDBs database. Legends on the right stand for colors of different host, isolates, and geographic information. Different colors stand for copy number of each virulence factors.

*Staphylococcus epidermidis* is the major colonization microorganisms in the human skin with complex human-bacterium and bacterium-bacterium interactions. We analyzed the genes (Figure 5, Table 1 and Supplementary Table S5) involved in resistance against AMPs that can inhibit the growth of most skin microorganism including *S. epidermidis*. We also identified 25 genes related to biofilm formation (7/17) and cell toxicity (8/17), which were significantly enriched in the blood and skin, were reported to assist the strain to survive on the skin surface (Joo and Otto, 2015). We also analyzed genes involved in bacterium-bacterium interactions. We found that the genes involved in short-chain fatty acids biosynthesis and extracellular proteases (e.g., Esp) had no difference among the isolates.

**TABLE 1:** *Staphylococcus epidermidis* resistance mechanisms that target antimicrobial peptides (AMPs).

Resistance Mechanism	Gene	Target AMPs	Functions	Enrichment
AMP sensing	*aps*SRX	Most cationic AMPs	3-Component sensor/regulator	–
	braSR/braDE/vraDE	Bacitracin, nisin		–
Phosphatidylglycerol lysylation	*mpr*F	Most cationic AMPs	Lysylation of membrane phospholipids	–
Teichoic acids alanylation	*dlt*ABCD	Most cationic AMPs	Alanylation of teichoic acids	Blood/Skin (*dlt*D)
Exopolymers	*ica*ADBC	HBD3, LL-37, DCD-1	Production of PNAG exopolysaccharide; IcaB *N*-acetylglucosamine deacetylase introduces positive charge	Blood(*ica*B)
	*cap*ABCD	HBD3, LL-37, DCD-1		
Extracellular proteases	*sep*A	LL-37	Degrades AMPs	–
	*esp*	LL-37a		–
ABC transporters	*vra*FG	Vancomycin, polymyxin B, colistin	Putative AMP exporter	–

## Discussion

*Staphylococcus epidermidis* is a coagulase-negative and Gram-positive staphylococcus that is part of the normal mucosa and skin microflora in humans and other mammals (Oh et al., 2016). It is the second leading cause of nosocomial infections (Ziebuhr et al., 2006). Although *S. epidermidis* is a saprophyte and an opportunistic pathogen with plenty of antibiotic resistance and virulence factors (Namvar et al., 2014), this natural skin colonizer plays a critical role in balancing the epithelial microflora (Otto, 2009, 2010). As an innocuous commensal microorganism, *S. epidermidis* has long been seen as an avirulent species. With the accumulation of genomic sequences, we can now further explore the genetic mechanisms of environmental adaptability of *S. epidermidis*, the evolution process during the outbreak, and the molecular biomarkers for clinical diagnosis (Otto, 2009; Didelot et al., 2012).

In our current pan-genome analysis, *S. epidermidis* had a relatively compact genome with a size of about 2.5 Mb, and yet almost 20% of this genome was in flux, exchanging with a large pool of various genes. These findings were similar to what had been reported by Conlan et al. (2012). The significant number of genes involved in mobilome makes horizontal gene transfer easier among *Staphylococcus* stains and lead to the increase of the “open” pan-genome (Palmer et al., 2010). Besides, mobile genetic elements, such as SCC*mec*, ACME, and plasmids, make the genome structure more unpredictable (Qin et al., 2016). High-resolution phylogenetic tree constructed from genome-wide SNPs revealed important details not seen by traditional multi-locus sequence typing (MLST) or single gene marker (16S rDNA). From the phylogenetic tree, we found the ST 2 isolates had an extremely short evolutionary distance from each other.

The genetic markers *mec*A and *ica*A, which are used to predict the AMR and biofilm phenotypes, have been shown to be enriched in hospital isolates than in non-hospital isolates; however, these markers have much less power to distinguish infection isolates from commensally available isolates that contaminate clinical specimens (Tolo et al., 2016). According to our enrichment analysis, we found it was possible to distinguish the strains of blood from those of skin, with 17 potential biomarkers related to biofilm formation and cell toxicity, demonstrating the potential for identifying risk genotypes. Whole genome sequencing has been proved to be a more powerful routine diagnostic tool than the traditional MLST or RT-PCR because it can rapidly identify the infection source and antibiotic resistance in an affordable manner (Rasko et al., 2011; Pankhurst et al., 2016). As more genetic data of *S. epidermidis* have been available and a new machine learning algorithm has been developed (Tolo et al., 2016), WGS may help to predict the infection isolation sources and antibiotic resistance in a quicker and more accurate manner. On the other hand, *S. epidermidis* has very complicated relationship with human and other bacteria. The prevalence of *S. epidermidis* gene content and other genetic features exhibited strain specificity, suggesting functional specialization to the niche. AMPs play an important role in providing immunity to bacterial colonization on human epithelia (Liu et al., 2020; Zhou et al., 2020). Recent research has shown that *Staphylococci* have multiple systems to combat AMP activity, including AMP sensor that can regulate the expressions of genes involved in AMP resistance depending on the presence of AMPs (Joo and Otto, 2015). We analyzed the distribution of gene involved in AMP resistance and found the enrichment was significant in blood and skin and variable among different strains, which may be the consequence of coevolution of human’s immune system. On the other side, *S. epidermidis* strains also can inhibit the growth of other bacterium to be dominant species on the skin surface. Serine protease Esp, which is secreted by *S. epidermidis*, has been found to be able to inhibit the biofilm formation of *S. aureus* and destroy pre-existing *S. aureus* biofilms (Iwase et al., 2010). Other mechanisms are also involved in fighting against pathogens and maintaining homeostasis (Otto et al., 2001; Wang et al., 2014). *S. epidermidis* was also found to be a reservoir of antibiotic resistance, with its virulence determinants shared with other more pathogenic species such as *S. aureus*, as demonstrated in previous studies (Conlan et al., 2012). In particular, SCC*mec*, ACME elements conferring β-lactam resistance, and other genes are transferred frequently between *Staphylococcus* strains, enabling rapid evolution and adaptation against antibiotic selection pressure and provide additional competitive advantage. For instance, type III of SCC*mec* carries a phenol soluble modulin *psm-mec*, which may affect the virulence of *S. aureus* (Qin et al., 2016). This provides strong support for pathogen carriage and increased infection risks elsewhere in the body, such as of methicillin-resistant *S. aureus* in the nares, as well as for the contextual microbiome affecting infection risk via HGT of pathogenicity reservoirs.

## Conclusion

Our current study provides information on the molecular characteristics of *S. epidermidis* strains isolated from different environments from all over the world. From a genomic perspective, the pan-genome analysis of the *S. epidermidis* reveals a high level of diversity among the generic and strain-specific genes and provides novel insights into the adaptation and evolution of *S. epidermidis* isolates as opportunistic, multidrug-resistant nosocomial pathogens. *S*. *epidermidis* strains from habitats are not equivalent and pathogenic sub-population acquired genetic elements and related phenotypes that promote infection. We identified 17 potential biomarkers related to biofilm formation and cell toxicity, which may help to distinct blood strains from skin stains. A better understanding of the mechanisms of gene transfer will help to control the epidemic of pan-drug-resistant *S. epidermidis* strains.

## Data Availability Statement

The original contributions generated in the study are included in the article/Supplementary Materials, further inquiries can be directed to the corresponding authors.

## Author Contributions

FS, HX, and HC designed the project. FS conceived the experiment and analyzed the genome data. RT, YY, HL, GS, YL, BH, XX, XC, and GZ interpreted the results and drafted the manuscript. All authors reviewed the manuscript.

## Conflict of Interest

The authors declare that the research was conducted in the absence of any commercial or financial relationships that could be construed as a potential conflict of interest.

## Footnotes

1 https://sourceforge.net/projects/ksnp/

2 https://sourceforge.net/projects/panoct/

3 http://kobas.cbi.pku.edu.cn/

## Supplementary Material

Supplementary Table 1 Basic information of all strains analyzed used in this study.

Supplementary Table 2 Result of clusters of orthologous group (COG) enrichment analysis across all strains.

Supplementary Table 3 Result of KEGG enrichment analysis across all strains.

Supplementary Table 4 Cluster of orthologous proteins produced by PanOCT.

Supplementary Table 5 Enrichment analysis about antibiotic resistances from different sources.

Supplementary Table 6 Enrichment analysis about genes related in biofilm formation.

## Abbreviations

SCC*mec*: staphylococcal chromosome cassette *mec*
AMPs: antimicrobial peptides
SNPs: single-nucleotide polymorphisms
COGs: clusters of orthologous groups
ST: sequence type
AMR: antimicrobial resistance.
